# Supplementary material for: Knowledge, Attitude, and Perception of Health Care Providers Providing Medication Therapy Management (MTM) Services to Older Adults in Saudi Arabia
Source: Healthcare (Basel). 2023 Nov 10;11(22):2936. doi: 10.3390/healthcare11222936 (PMC10671085; doi:10.3390/healthcare11222936)
Supplement: Supplementary file 1 [file healthcare-11-02936-s001.zip › healthcare-2678355-supplementary.pdf]

Knowledge, Attitude and Perception of health care providers providing Medication Therapy Management (MTM) service to older adults

Dear Participants,

A 5th year students from College of Clinical Pharmacy at Imam Abdulrahman Bin Faisal University in Dammam, Saudi Arabia is aiming to conduct a study to investigate the level of knowledge, attitude, and Barriers of health care providers about medication therapy management services provided to older adult population in the Kingdom of Saudi Arabia. Participating in this survey is completely voluntary and the principal investigator is not collecting any personal information nor storing any. This study went through an ethical review from the IRB committee at IAU and approved as an exempt study. If you have any question, please don't hesitate to contact the principal investigator Dr. Fawaz Alotaibi via fmalotaibi@iau.edu.sa. The estimating time to complete the survey is 10 to 15 minutes, your participation is very much appreciated. I agree and confirm that I am a primary care physician or a community pharmacist eligible to participate in this study. By choosing Agree to participate; bellow you understand that the participation in this research is voluntary and I you are free to refuse to participate and withdraw from the research at any time.

1. I agree to participate in this research.

This section is to assess the knowledge regarding MTM services.MTM means: Medication Therapy ManagementHealth care provider means: any Primary Care physician or Pharmacist working with older adults population in a direct or in an indirect way

1- MTM is defined as service or group of services that: optimize therapeutic outcomes for individual patients .

1. True
2. False
3. I dont know

2- The core elements of MTM service are Medication Therapy Review (MTR), Personal Medication Record (PMR), Medication Related Action Plan (MAP), Intervention or Referral , Documentation and Follow Up.

1. True
2. False
3. I dont know

3- Medication therapy management services have three goals which are: to improve the understanding medication uses, medication adherence and detection of medication related problems.

1. True
2. False
3. I dont know

4- Any patient who uses prescription and nonprescription medications, herbal products or other dietary supplements could potentially benefit from MTM service.

1. True
2. False
3. I dont know

5- Primary role of MTM service is aid with adherence and disease state management.

1. True
2. False
3. I dont know

Knowledge, Attitude and Perception of health care providers providing Medication Therapy Management (MTM) service to older adults

This section measures the Attitude and Barriers regarding providing MTM

Please select the best choice regarding every provided statement.

	Strongly Agree	Agree	Neutral	Disagree	Strongly Disagree
If MTM service will be implemented in the future, would you like to be an MTM service provider ?	❑	❑	❑	❑	❑
Do you think that providing MTM services is only hospital-based program ?	❑	❑	❑	❑	❑
Do you think that elderly directed MTM services subscription programs are worthy of initiation in primary care ?	❑	❑	❑	❑	❑
Do you think implementing elderly directed MTM service in the future is important to improve their quality of life ?	❑	❑	❑	❑	❑
Are you interested in learning more information about elderly directed MTM service ?	❑	❑	❑	❑	❑
Is online education a good way to provide training about MTM ?	❑	❑	❑	❑	❑
Do you prefer live workshops as a training method about MTM ?	❑	❑	❑	❑	❑
I am willing to provide phone counselling about MTM to the elderly patients ?	❑	❑	❑	❑	❑
I am willing to provide home visits to deliver the MTM services to the elderly centers/homes ?	❑	❑	❑	❑	❑
In your current practice, do you think that you spend enough time counselling your elderly patients ?	❑	❑	❑	❑	❑
Do you think that you will have enough time to apply MTM service in the future ?	❑	❑	❑	❑	❑
Does your pharmacy or the place that you work at currently have a private consultation room for the patients ?	❑	❑	❑	❑	❑
Do you usually access (online or hard copies) most updated geriatric directed treatment guidelines available for diseases such as AGS Beers Criteria® ..etc ?	❑	❑	❑	❑	❑
Do you have an easy access (online or hard copies) to the geriatric guidelines to manage your elderly patients ?	❑	❑	❑	❑	❑
Lack of training in MTM services delivery is one of the potential barriers regarding applying elderly directed MTM service in the future ?	❑	❑	❑	❑	❑
Do you think that applying elderly directed MTM services need high budget ?	❑	❑	❑	❑	❑
I believe that geriatrics should receive more care than any other group of patients ?	❑	❑	❑	❑	❑
I feel confident in my communication skills while counselling an elderly patients ?	❑	❑	❑	❑	❑
I have the required competencies to deliver MTM services to the elderly patients ?	❑	❑	❑	❑	❑
I believe that geriatrics should receive more education than any other group of patients ?	❑	❑	❑	❑	❑

Knowledge, Attitude and Perception of health care providers providing Medication Therapy Management (MTM) service to older adults

Demographic Information:

1- Age (years)
1. 20-29
2. 30-39
3. 40-49
4. above 50

2-Gender
1. Male
2. Female

3- What is the latest degree you have accomplished?
1. - Bachelor's degree
2. - Master's degree
3. - PhD Degree
4. - Residency and post bachelor's degree

4- What is your specialty?
1. - Pharmacist (Community pharmacist)
2. - Medical Doctor

5-Years of experience?
1. - less than 2 years
2. - 2 -5 years
3. - 6-10 years
4. - 11-15 years
5. - more than 15 years

6-The region in which you work in Saudi Arabia?
1. Central Region
2. Eastern Region
3. Western Region
4. Northern Region
5. Southern Region

7- Do you have assistance provided to you in your work area to share the workload of providing care for your patients (nurse , technician or work peer etc..)
1. Yes
2. No

Knowledge, Attitude and Perception of health care providers providing Medication Therapy Management (MTM) service to older adults

8-Hours worked/week

1. - 40 hours or less
2. - More than 40 hours

9-The estimate number of prescription medications dispensed per day for the elderly patients?

1. less than 50
2. 50-300
3. more than 300
4. Not sure
